# 3,3-Bis[(4-meth­oxy­phen­yl)sulfan­yl]-1-methyl­piperidin-2-one

**DOI:** 10.1107/S1600536812025202

**Published:** 2012-06-13

**Authors:** Ignez Caracelli, Paulo R. Olivato, Carlos R. Cerqueira, Jean M. M. Santos, Seik Weng Ng, Edward R. T. Tiekink

**Affiliations:** a BioMat - Departmento de Física, Universidade Federal de São Carlos, 13565-905 São Carlos-SP, Brazil; b Chemistry Institute, Universidade de São Paulo, 05508-000 São Paulo-SP, Brazil; c Department of Chemistry, University of Malaya, 50603 Kuala Lumpur, Malaysia, Chemistry Department, Faculty of Science, King Abdulaziz University, PO Box 80203 Jeddah, Saudi Arabia; d Department of Chemistry, University of Malaya, 50603 Kuala Lumpur, Malaysia

## Abstract

The piperidone ring in the title compound, C_20_H_23_NO_3_S_2_, has a distorted half-chair conformation with the central methyl­ene atom of the propyl fragment lying 0.696 (1) Å out of the plane defined by the other five atoms (r.m.s. deviation = 0.071 Å). One of the S-bound phenyl rings is almost perpendicular to the mean plane through the piperidone ring, whereas the other is splayed [dihedral angles = 71.95 (6) and 38.42 (6)°]. In the crystal, C—H⋯O and C—H⋯π inter­actions lead to the formation of supra­molecular layers in the *ab* plane.

## Related literature

For background to β-thio­carbonyl compounds, see: Vinhato *et al.* (2011 ▶); Olivato *et al.* (2009 ▶). For related structures, see: Caracelli *et al.* (2012 ▶); Zukerman-Schpector *et al.* (2010 ▶, 2011 ▶). For ring conformational analysis, see: Cremer & Pople (1975 ▶). For the synthesis, see: Hashmat & McDermott, (2002 ▶); Zoretic & Soja (1976 ▶).
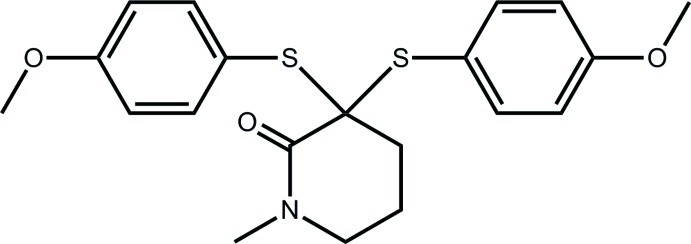


## Experimental

### 

#### Crystal data

C_20_H_23_NO_3_S_2_
*M* *_r_* = 389.53
Monoclinic, 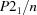

*a* = 8.5802 (1) Å
*b* = 9.4744 (1) Å
*c* = 23.3732 (2) Å
β = 91.018 (1)°
*V* = 1899.76 (3) Å^3^
*Z* = 4
Cu *K*α radiation
μ = 2.70 mm^−1^
*T* = 100 K
0.30 × 0.25 × 0.20 mm

#### Data collection

Agilent SuperNova Dual (Cu at zero) Atlas detector diffractometer
Absorption correction: multi-scan (*CrysAlis PRO*; Agilent, 2011 ▶) *T*_min_ = 0.498, *T*_max_ = 0.614
7011 measured reflections
3763 independent reflections
3484 reflections with *I* > 2σ(*I*)
*R*_int_ = 0.016

#### Refinement

*R*[*F* ^2^ > 2σ(*F* ^2^)] = 0.029
*wR*(*F* ^2^) = 0.076
*S* = 1.06
3763 reflections
238 parameters
H-atom parameters constrained
Δρ_max_ = 0.25 e Å^−3^
Δρ_min_ = −0.35 e Å^−3^

### 

Data collection: *CrysAlis PRO* (Agilent, 2011 ▶); cell refinement: *CrysAlis PRO*; data reduction: *CrysAlis PRO*; program(s) used to solve structure: *SIR92* (Altomare *et al.*, 1999 ▶); program(s) used to refine structure: *SHELXL97* (Sheldrick, 2008 ▶); molecular graphics: *ORTEP-3* (Farrugia, 1997 ▶), *DIAMOND* (Brandenburg, 2006 ▶) and *MarvinSketch* (Chemaxon, 2009 ▶); software used to prepare material for publication: *publCIF* (Westrip, 2010 ▶).

## Supplementary Material

Crystal structure: contains datablock(s) global, I. DOI: 10.1107/S1600536812025202/su2445sup1.cif

Structure factors: contains datablock(s) I. DOI: 10.1107/S1600536812025202/su2445Isup2.hkl

Supplementary material file. DOI: 10.1107/S1600536812025202/su2445Isup3.cml

Additional supplementary materials:  crystallographic information; 3D view; checkCIF report

## Figures and Tables

**Table 1:** Hydrogen-bond geometry (Å, °) *Cg*1 is the centroid of the C7–C12 ring.

*D*—H⋯*A*	*D*—H	H⋯*A*	*D*⋯*A*	*D*—H⋯*A*
C11—H11⋯O1^i^	0.95	2.50	3.4211 (16)	165
C9—H9⋯O3^ii^	0.95	2.48	3.3473 (17)	151
C6—H6b⋯*Cg*1^ii^	0.98	2.93	3.5232 (17)	120

Symmetry codes: (i) 
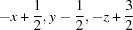
; (ii) 
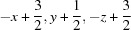
.

**Fig. 1.**
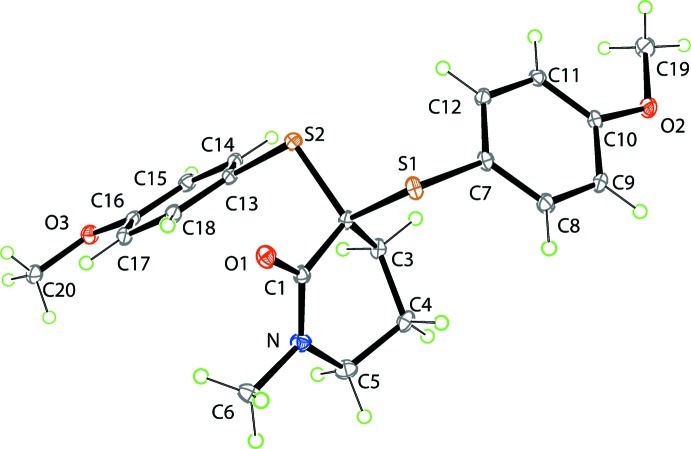
The molecular structure of (I) showing the atom labelling and displacement ellipsoids at the 50% probability level (arbitrary spheres for the H atoms).

**Fig. 2.**
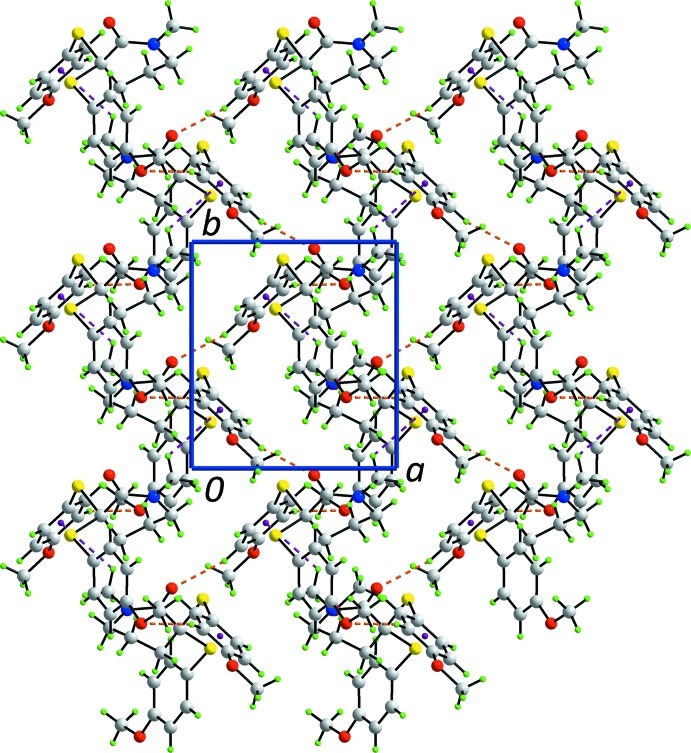
A view of a supramolecular layer in the ab plane in (I). The C—H···O and C—H···π interactions are shown as orange and purple dashed lines, respectively.

**Fig. 3.**
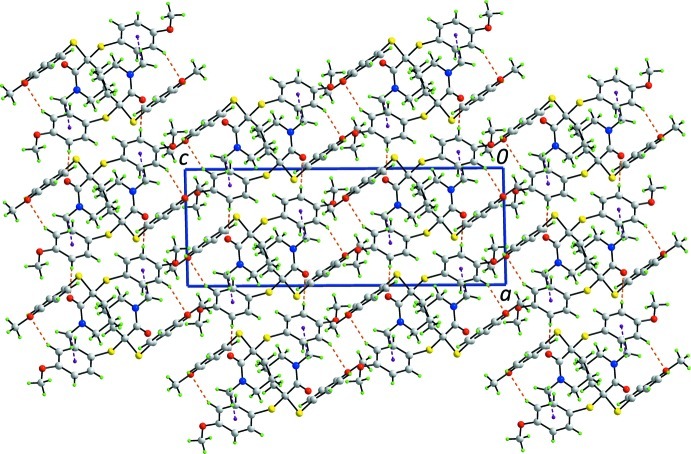
A view in projection down the b axis of the unit-cell contents for (I). The C—H···O and C—H···π interactions are shown as orange and purple dashed lines, respectively.

## supplementary crystallographic information

### Comment

As part of our on-going research on the conformational behaviour and electronic interactions in β-thio-carbonyl (Vinhato *et al.*, 2011) and β-bis-thio-carbonyl compounds, *e.g. N*-methoxy-*N*-methyl-2-[(4'-substituted) phenylthio]propanamides and 3,3-bis[(4'- substituted) phenylthio]-1-methyl-2-piperidones, using spectroscopic, theoretical and X-ray diffraction methods (Olivato *et al.*, 2009; Zukerman-Schpector *et al.*, 2010, 2011; Caracelli *et al.*, 2012), the title compound, (I), was synthesized and its crystal structure is reported herein.

In (I), Fig. 1, the piperidone ring is in a distorted half-chair conformation with the C4 atom lying 0.696 (1) Å out of the plane defined by the other five atoms (r.m.s. deviation = 0.071 Å). The ring puckering parameters are: q_2_ = 0.4418 (14) Å, q_3_ = -0.2835 (14) Å, QT = 0.5249 (15) Å, φ_2_ = 38.90 (18) ° (Cremer & Pople, 1975). The S2-bound phenyl ring is almost perpendicular to the mean plane through the piperidone ring [dihedral angle = 71.95 (6) °] whereas the S1-bond phenyl ring makes dihedral angles of 38.42 (6) and 69.65 (6)° with the mean planes of the piperidone and S2-bound phenyl rings, respectively. The overall molecular conformation observed for (I) resembles that seen in the species without methoxy groups in the 4-positions of the benzene rings (Caracelli *et al.*, 2012).

The crystal packing of (I) features C—H···O and C—H···π interactions that lead to the formation of supramolecular layers (Table 1 and Fig. 2). These stack along the *c* axis without specific intermolecular interactions between them (Fig. 3).

### Experimental

4-Methoxylthiophenol (4.9 ml, 40 mmol) was oxidized with bromine (1.1 ml, 20 mmol) in dichloromethane (250 ml) on hydrated silica gel support (25 g of SiO_2_ and 12 ml of water) to give 4-methoxylphenyl disulfide (4.8 g, yield = 85%). A white solid was obtained after filtration and evaporation without further purification (Hashmat & McDermott, 2002). 1-Methyl-2-piperidinone (1.9 g, 17 mmol) was added drop-wise to a cooled (195 K) solution of hexamethylphosphoramide (HMPA) (3.1 ml, 17 mmol), diisopropylamine (2.4 ml, 17 mmol) and butyllithium (11.2 ml, 1.52 mol *L*^-1^ hexane solution) in THF (50 ml). After 20 minutes, 4-methoxylphenyl disulfide (4.8 g, 17 mmol) dissolved in THF (15 ml) was added drop-wise to the enolate solution (Zoretic & Soja, 1976). After the mixture was stirred for 4 h at 195 K, water (100 ml) was added at room temperature and extraction with dichloromethane was performed. The organic layer was dried over anhydrous sodium sulfate. After evaporation of solvent, a crude solid was obtained. Purification through flash chromatography with a solution of hexane and ethyl acetate in a 7:3 ratio give the pure product (2.5 g, yield = 37%). Colourless crystals of (I), suitable for X-ray diffraction analysis, were obtained by vapour diffusion of *n*-hexane into a chloroform solution held at 283 K; *M*.pt: 430–431 K. Analysis found: C 61.68, H 5.66, N 3.55%. C_20_H_23_ONS_2_ requires: C 61.66, H 5.95, N 3.60%. Spectroscopic data for compound (I) are given in the archived CIF.

### Refinement

The H atoms were included in calculated positions (C—H = 0.95–0.99 Å) and refined as riding with *U_iso_*(H) = 1.2–1.5*U_eq_*(C).

### Crystal data

**Table:**

C_20_H_23_NO_3_S_2_	*F*(000) = 824
*M**_r_* = 389.53	*D*_x_ = 1.362 Mg m^−^^3^
Monoclinic, *P*2_1_/*n*	Cu *K*α radiation, λ = 1.54184 Å
Hall symbol: -P 2yn	Cell parameters from 4644 reflections
*a* = 8.5802 (1) Å	θ = 3.8–74.3°
*b* = 9.4744 (1) Å	µ = 2.70 mm^−^^1^
*c* = 23.3732 (2) Å	*T* = 100 K
β = 91.018 (1)°	Prism, colourless
*V* = 1899.76 (3) Å^3^	0.30 × 0.25 × 0.20 mm
*Z* = 4	

### Data collection

**Table:**

Agilent SuperNova Dual (Cu at zero) Atlas detector diffractometer	3763 independent reflections
Radiation source: fine-focus sealed tube	3484 reflections with *I* > 2σ(*I*)
Graphite monochromator	*R*_int_ = 0.016
Detector resolution: 10.4041 pixels mm^-1^	θ_max_ = 74.5°, θ_min_ = 3.8°
ω scans	*h* = −9→10
Absorption correction: multi-scan (*CrysAlis PRO*; Agilent, 2011)	*k* = −7→11
*T*_min_ = 0.498, *T*_max_ = 0.614	*l* = −28→28
7011 measured reflections	

### Refinement

**Table:**

Refinement on *F*^2^	Primary atom site location: structure-invariant direct methods
Least-squares matrix: full	Secondary atom site location: difference Fourier map
*R*[*F*^2^ > 2σ(*F*^2^)] = 0.029	Hydrogen site location: inferred from neighbouring sites
*wR*(*F*^2^) = 0.076	H-atom parameters constrained
*S* = 1.06	*w* = 1/[σ^2^(*F*_o_^2^) + (0.0385*P*)^2^ + 0.7353*P*] where *P* = (*F*_o_^2^ + 2*F*_c_^2^)/3
3763 reflections	(Δ/σ)_max_ < 0.001
238 parameters	Δρ_max_ = 0.25 e Å^−^^3^
0 restraints	Δρ_min_ = −0.35 e Å^−^^3^

### Special details

**Table:**

Experimental. Spectroscopic data for compound (I):IR (cm^-1^): ν(C=O) 1662. NMR (CDCl_3_, p.p.m.): δ 1.86–1.90 (2*H*, multiplet), 1.93–1.95 (2*H*, multiplet), 2.89 (3*H*, singlet), 3.12–3.14 (2*H*, triplet, J = 6.0 Hz), 3.82 (6*H*, singlet) 6.84–6.87 (4*H*, multiplet, Aryl-H), 7.53–7.55 (4*H*, multiplet, Aryl-H).
Geometry. All s.u.'s (except the s.u. in the dihedral angle between two l.s. planes) are estimated using the full covariance matrix. The cell s.u.'s are taken into account individually in the estimation of s.u.'s in distances, angles and torsion angles; correlations between s.u.'s in cell parameters are only used when they are defined by crystal symmetry. An approximate (isotropic) treatment of cell s.u.'s is used for estimating s.u.'s involving l.s. planes.
Refinement. Refinement of *F*^2^ against ALL reflections. The weighted *R*-factor *wR* and goodness of fit *S* are based on *F*^2^, conventional *R*-factors *R* are based on *F*, with *F* set to zero for negative *F*^2^. The threshold expression of *F*^2^ > σ(*F*^2^) is used only for calculating *R*-factors(gt) *etc*. and is not relevant to the choice of reflections for refinement. *R*-factors based on *F*^2^ are statistically about twice as large as those based on *F*, and *R*- factors based on ALL data will be even larger.

### Fractional atomic coordinates and isotropic or equivalent isotropic displacement parameters (Å^2^)

**Table:**

	*x*	*y*	*z*	*U*_iso_*/*U*_eq_	
C1	0.65880 (15)	0.88587 (14)	0.83750 (5)	0.0156 (3)	
C2	0.55775 (15)	0.79082 (13)	0.79859 (5)	0.0149 (3)	
C3	0.64992 (16)	0.68485 (14)	0.76382 (6)	0.0171 (3)	
H3A	0.6783	0.6030	0.7882	0.021*	
H3B	0.5846	0.6503	0.7314	0.021*	
C4	0.79668 (16)	0.75248 (15)	0.74115 (6)	0.0201 (3)	
H4A	0.8519	0.6850	0.7163	0.024*	
H4B	0.7693	0.8368	0.7181	0.024*	
C5	0.90077 (17)	0.79433 (16)	0.79111 (6)	0.0234 (3)	
H5A	0.9456	0.7084	0.8090	0.028*	
H5B	0.9880	0.8526	0.7771	0.028*	
C6	0.90807 (18)	0.95375 (17)	0.87618 (7)	0.0280 (3)	
H6A	0.8835	0.9215	0.9148	0.042*	
H6B	0.8841	1.0545	0.8726	0.042*	
H6C	1.0191	0.9385	0.8692	0.042*	
C7	0.39879 (16)	0.82787 (14)	0.69129 (5)	0.0164 (3)	
C8	0.48089 (16)	0.85797 (15)	0.64178 (6)	0.0197 (3)	
H8	0.5623	0.9259	0.6429	0.024*	
C9	0.44427 (17)	0.78940 (15)	0.59104 (6)	0.0205 (3)	
H9	0.5007	0.8103	0.5575	0.025*	
C10	0.32476 (16)	0.68970 (14)	0.58905 (5)	0.0166 (3)	
C11	0.24152 (15)	0.65820 (14)	0.63809 (6)	0.0161 (3)	
H11	0.1602	0.5902	0.6369	0.019*	
C12	0.27956 (15)	0.72801 (14)	0.68886 (6)	0.0169 (3)	
H12	0.2232	0.7072	0.7225	0.020*	
C13	0.52232 (15)	0.59131 (14)	0.88711 (6)	0.0167 (3)	
C14	0.52403 (16)	0.44562 (15)	0.87749 (6)	0.0192 (3)	
H14	0.4702	0.4078	0.8451	0.023*	
C15	0.60328 (16)	0.35581 (15)	0.91466 (6)	0.0208 (3)	
H15	0.6035	0.2570	0.9078	0.025*	
C16	0.68264 (15)	0.41136 (15)	0.96214 (6)	0.0181 (3)	
C17	0.68445 (16)	0.55632 (15)	0.97184 (6)	0.0185 (3)	
H17	0.7409	0.5942	1.0036	0.022*	
C18	0.60273 (16)	0.64528 (15)	0.93459 (6)	0.0184 (3)	
H18	0.6018	0.7440	0.9416	0.022*	
C19	0.18049 (18)	0.52401 (17)	0.53274 (6)	0.0269 (3)	
H19A	0.1766	0.4858	0.4938	0.040*	
H19B	0.0803	0.5681	0.5414	0.040*	
H19C	0.2015	0.4475	0.5600	0.040*	
C20	0.84281 (17)	0.36600 (17)	1.04494 (6)	0.0247 (3)	
H20A	0.8842	0.2863	1.0672	0.037*	
H20B	0.7759	0.4237	1.0692	0.037*	
H20C	0.9294	0.4236	1.0312	0.037*	
N	0.81502 (13)	0.87439 (12)	0.83427 (5)	0.0190 (2)	
O1	0.59763 (11)	0.96898 (10)	0.87087 (4)	0.0203 (2)	
O2	0.30155 (12)	0.62711 (11)	0.53720 (4)	0.0226 (2)	
O3	0.75382 (12)	0.31405 (11)	0.99716 (4)	0.0225 (2)	
S1	0.44547 (4)	0.92077 (3)	0.755059 (13)	0.01775 (9)	
S2	0.40881 (4)	0.70322 (4)	0.841568 (14)	0.01771 (9)	

### Atomic displacement parameters (Å^2^)

**Table:**

	*U*^11^	*U*^22^	*U*^33^	*U*^12^	*U*^13^	*U*^23^
C1	0.0190 (6)	0.0141 (6)	0.0138 (6)	−0.0017 (5)	0.0000 (5)	0.0019 (5)
C2	0.0174 (6)	0.0146 (6)	0.0127 (6)	−0.0002 (5)	0.0004 (5)	0.0012 (5)
C3	0.0229 (7)	0.0139 (6)	0.0146 (6)	0.0017 (5)	0.0006 (5)	−0.0001 (5)
C4	0.0243 (7)	0.0181 (7)	0.0181 (7)	0.0040 (6)	0.0066 (5)	0.0011 (5)
C5	0.0194 (7)	0.0217 (7)	0.0292 (8)	0.0019 (6)	0.0043 (6)	−0.0015 (6)
C6	0.0228 (7)	0.0278 (8)	0.0330 (8)	−0.0062 (6)	−0.0067 (6)	−0.0042 (7)
C7	0.0195 (6)	0.0153 (6)	0.0142 (6)	0.0019 (5)	−0.0028 (5)	0.0010 (5)
C8	0.0218 (7)	0.0185 (7)	0.0188 (7)	−0.0047 (5)	−0.0028 (5)	0.0046 (5)
C9	0.0229 (7)	0.0249 (7)	0.0136 (6)	−0.0032 (6)	0.0013 (5)	0.0053 (5)
C10	0.0189 (6)	0.0169 (6)	0.0139 (6)	0.0023 (5)	−0.0025 (5)	0.0014 (5)
C11	0.0148 (6)	0.0164 (6)	0.0170 (6)	0.0006 (5)	−0.0014 (5)	0.0018 (5)
C12	0.0164 (6)	0.0192 (7)	0.0151 (6)	0.0022 (5)	0.0013 (5)	0.0023 (5)
C13	0.0170 (6)	0.0192 (6)	0.0141 (6)	−0.0030 (5)	0.0028 (5)	0.0022 (5)
C14	0.0214 (7)	0.0215 (7)	0.0149 (6)	−0.0061 (6)	0.0018 (5)	−0.0002 (5)
C15	0.0236 (7)	0.0176 (7)	0.0213 (7)	−0.0041 (5)	0.0051 (5)	0.0002 (5)
C16	0.0162 (6)	0.0218 (7)	0.0165 (6)	−0.0004 (5)	0.0044 (5)	0.0048 (5)
C17	0.0198 (6)	0.0221 (7)	0.0136 (6)	−0.0032 (5)	0.0013 (5)	−0.0002 (5)
C18	0.0216 (7)	0.0180 (7)	0.0157 (6)	−0.0024 (5)	0.0029 (5)	−0.0007 (5)
C19	0.0302 (8)	0.0282 (8)	0.0223 (7)	−0.0073 (7)	−0.0015 (6)	−0.0068 (6)
C20	0.0228 (7)	0.0324 (8)	0.0187 (7)	−0.0002 (6)	0.0004 (5)	0.0074 (6)
N	0.0175 (6)	0.0182 (6)	0.0212 (6)	−0.0028 (5)	−0.0003 (4)	−0.0020 (5)
O1	0.0235 (5)	0.0196 (5)	0.0178 (5)	0.0006 (4)	−0.0007 (4)	−0.0053 (4)
O2	0.0264 (5)	0.0271 (5)	0.0143 (5)	−0.0055 (4)	−0.0002 (4)	−0.0029 (4)
O3	0.0241 (5)	0.0221 (5)	0.0213 (5)	0.0003 (4)	−0.0003 (4)	0.0063 (4)
S1	0.02349 (18)	0.01403 (16)	0.01560 (16)	0.00191 (12)	−0.00321 (12)	−0.00080 (11)
S2	0.01626 (16)	0.02082 (17)	0.01604 (16)	−0.00271 (12)	0.00022 (12)	0.00262 (12)

### Geometric parameters (Å, º)

**Table:**

C1—O1	1.2323 (16)	C10—O2	1.3607 (16)
C1—N	1.3483 (17)	C10—C11	1.3936 (18)
C1—C2	1.5368 (18)	C11—C12	1.3922 (18)
C2—C3	1.5220 (18)	C11—H11	0.9500
C2—S2	1.8376 (13)	C12—H12	0.9500
C2—S1	1.8558 (13)	C13—C18	1.3935 (18)
C3—C4	1.5168 (19)	C13—C14	1.3986 (19)
C3—H3A	0.9900	C13—S2	1.7801 (14)
C3—H3B	0.9900	C14—C15	1.386 (2)
C4—C5	1.510 (2)	C14—H14	0.9500
C4—H4A	0.9900	C15—C16	1.395 (2)
C4—H4B	0.9900	C15—H15	0.9500
C5—N	1.4700 (18)	C16—O3	1.3694 (16)
C5—H5A	0.9900	C16—C17	1.392 (2)
C5—H5B	0.9900	C17—C18	1.3923 (19)
C6—N	1.4610 (18)	C17—H17	0.9500
C6—H6A	0.9800	C18—H18	0.9500
C6—H6B	0.9800	C19—O2	1.4283 (18)
C6—H6C	0.9800	C19—H19A	0.9800
C7—C12	1.3939 (19)	C19—H19B	0.9800
C7—C8	1.3947 (19)	C19—H19C	0.9800
C7—S1	1.7708 (13)	C20—O3	1.4290 (17)
C8—C9	1.3835 (19)	C20—H20A	0.9800
C8—H8	0.9500	C20—H20B	0.9800
C9—C10	1.394 (2)	C20—H20C	0.9800
C9—H9	0.9500		
			
O1—C1—N	121.47 (12)	C11—C10—C9	120.33 (12)
O1—C1—C2	120.46 (12)	C12—C11—C10	118.92 (12)
N—C1—C2	118.07 (11)	C12—C11—H11	120.5
C3—C2—C1	114.17 (11)	C10—C11—H11	120.5
C3—C2—S2	111.53 (9)	C11—C12—C7	121.19 (12)
C1—C2—S2	109.37 (9)	C11—C12—H12	119.4
C3—C2—S1	114.48 (9)	C7—C12—H12	119.4
C1—C2—S1	102.57 (8)	C18—C13—C14	118.93 (13)
S2—C2—S1	103.89 (7)	C18—C13—S2	121.03 (11)
C4—C3—C2	110.50 (11)	C14—C13—S2	119.92 (11)
C4—C3—H3A	109.5	C15—C14—C13	120.77 (13)
C2—C3—H3A	109.5	C15—C14—H14	119.6
C4—C3—H3B	109.5	C13—C14—H14	119.6
C2—C3—H3B	109.5	C14—C15—C16	119.63 (13)
H3A—C3—H3B	108.1	C14—C15—H15	120.2
C5—C4—C3	108.91 (11)	C16—C15—H15	120.2
C5—C4—H4A	109.9	O3—C16—C17	124.28 (13)
C3—C4—H4A	109.9	O3—C16—C15	115.33 (12)
C5—C4—H4B	109.9	C17—C16—C15	120.38 (13)
C3—C4—H4B	109.9	C16—C17—C18	119.43 (13)
H4A—C4—H4B	108.3	C16—C17—H17	120.3
N—C5—C4	111.70 (11)	C18—C17—H17	120.3
N—C5—H5A	109.3	C17—C18—C13	120.84 (13)
C4—C5—H5A	109.3	C17—C18—H18	119.6
N—C5—H5B	109.3	C13—C18—H18	119.6
C4—C5—H5B	109.3	O2—C19—H19A	109.5
H5A—C5—H5B	107.9	O2—C19—H19B	109.5
N—C6—H6A	109.5	H19A—C19—H19B	109.5
N—C6—H6B	109.5	O2—C19—H19C	109.5
H6A—C6—H6B	109.5	H19A—C19—H19C	109.5
N—C6—H6C	109.5	H19B—C19—H19C	109.5
H6A—C6—H6C	109.5	O3—C20—H20A	109.5
H6B—C6—H6C	109.5	O3—C20—H20B	109.5
C12—C7—C8	119.09 (12)	H20A—C20—H20B	109.5
C12—C7—S1	121.77 (10)	O3—C20—H20C	109.5
C8—C7—S1	119.12 (10)	H20A—C20—H20C	109.5
C9—C8—C7	120.31 (13)	H20B—C20—H20C	109.5
C9—C8—H8	119.8	C1—N—C6	116.94 (12)
C7—C8—H8	119.8	C1—N—C5	126.17 (12)
C8—C9—C10	120.16 (13)	C6—N—C5	116.84 (12)
C8—C9—H9	119.9	C10—O2—C19	117.24 (11)
C10—C9—H9	119.9	C16—O3—C20	117.50 (11)
O2—C10—C11	124.76 (12)	C7—S1—C2	103.84 (6)
O2—C10—C9	114.89 (12)	C13—S2—C2	102.56 (6)
			
O1—C1—C2—C3	−174.73 (12)	O3—C16—C17—C18	−177.42 (12)
N—C1—C2—C3	4.69 (17)	C15—C16—C17—C18	1.8 (2)
O1—C1—C2—S2	−48.98 (15)	C16—C17—C18—C13	−1.5 (2)
N—C1—C2—S2	130.43 (11)	C14—C13—C18—C17	0.4 (2)
O1—C1—C2—S1	60.84 (14)	S2—C13—C18—C17	176.48 (10)
N—C1—C2—S1	−119.75 (11)	O1—C1—N—C6	6.26 (19)
C1—C2—C3—C4	−40.74 (15)	C2—C1—N—C6	−173.14 (12)
S2—C2—C3—C4	−165.34 (9)	O1—C1—N—C5	−171.00 (13)
S1—C2—C3—C4	77.07 (12)	C2—C1—N—C5	9.59 (19)
C2—C3—C4—C5	63.62 (14)	C4—C5—N—C1	13.72 (19)
C3—C4—C5—N	−49.55 (15)	C4—C5—N—C6	−163.55 (12)
C12—C7—C8—C9	0.0 (2)	C11—C10—O2—C19	1.48 (19)
S1—C7—C8—C9	−178.44 (11)	C9—C10—O2—C19	179.98 (13)
C7—C8—C9—C10	0.1 (2)	C17—C16—O3—C20	−3.66 (19)
C8—C9—C10—O2	−178.68 (12)	C15—C16—O3—C20	177.13 (12)
C8—C9—C10—C11	−0.1 (2)	C12—C7—S1—C2	77.37 (12)
O2—C10—C11—C12	178.54 (12)	C8—C7—S1—C2	−104.27 (11)
C9—C10—C11—C12	0.1 (2)	C3—C2—S1—C7	30.09 (11)
C10—C11—C12—C7	−0.1 (2)	C1—C2—S1—C7	154.33 (8)
C8—C7—C12—C11	0.1 (2)	S2—C2—S1—C7	−91.77 (7)
S1—C7—C12—C11	178.42 (10)	C18—C13—S2—C2	77.44 (12)
C18—C13—C14—C15	0.5 (2)	C14—C13—S2—C2	−106.48 (11)
S2—C13—C14—C15	−175.69 (10)	C3—C2—S2—C13	61.21 (10)
C13—C14—C15—C16	−0.2 (2)	C1—C2—S2—C13	−66.04 (10)
C14—C15—C16—O3	178.31 (12)	S1—C2—S2—C13	−174.98 (6)
C14—C15—C16—C17	−0.9 (2)		

### Hydrogen-bond geometry (Å, º)

Cg1 is the centroid of the C7–C12 ring.

**Table:**

*D*—H···*A*	*D*—H	H···*A*	*D*···*A*	*D*—H···*A*
C11—H11···O1^i^	0.95	2.50	3.4211 (16)	165
C9—H9···O3^ii^	0.95	2.48	3.3473 (17)	151
C6—H6b···*Cg*1^ii^	0.98	2.93	3.5232 (17)	120

Symmetry codes: (i) −*x*+1/2, *y*−1/2, −*z*+3/2; (ii) −*x*+3/2, *y*+1/2, −*z*+3/2.

## References

[bb1] Agilent (2011). *CrysAlis PRO* Agilent Technologies, Yarnton, England.

[bb2] Altomare, A., Burla, M. C., Camalli, M., Cascarano, G. L., Giacovazzo, C., Guagliardi, A., Moliterni, A. G. G., Polidori, G. & Spagna, R. (1999). *J. Appl. Cryst.* **32**, 115–119.

[bb3] Brandenburg, K. (2006). *DIAMOND* Crystal Impact GbR, Bonn, Germany.

[bb4] Caracelli, I., Olivato, P. R., Cerqueira Jr, C. R., Santos, J. M. M., Ng, S. W. & Tiekink, E. R. T. (2012). *Acta Cryst.* E**68**, o1793–o1794. 10.1107/S1600536812021277 PMC3379371 22719569

[bb5] Chemaxon (2009). *MarvinSketch* *www.chemaxon.com*

[bb6] Cremer, D. & Pople, J. A. (1975). *J. Am. Chem. Soc.* **97**, 1354–1358.

[bb7] Farrugia, L. J. (1997). *J. Appl. Cryst.* **30**, 565.

[bb8] Hashmat, A. M. & McDermott, M. (2002). *Tetrahedron Lett.* **43**, 6271–6273.

[bb9] Olivato, P. R., Domingues, N. L. C., Mondino, M. G., Tormena, C. F., Rittner, R. & Dal Colle, M. (2009). *J. Mol. Struct.* **920**, 393–400.

[bb10] Sheldrick, G. M. (2008). *Acta Cryst.* A**64**, 112–122. 10.1107/S0108767307043930 18156677

[bb11] Vinhato, E., Olivato, P. R., Rodrigues, A., Zukerman-Schpector, J. & Dal Colle, M. (2011). *J. Mol. Struct.* **1002**, 97–106.

[bb12] Westrip, S. P. (2010). *J. Appl. Cryst.* **43**, 920–925.

[bb13] Zoretic, P. A. & Soja, P. (1976). *J. Org. Chem.* **41**, 3587–3589.

[bb14] Zukerman-Schpector, J., De Simone, C. A., Olivato, P. R., Cerqueira, C. R., Santos, J. M. M. & Tiekink, E. R. T. (2010). *Acta Cryst.* E**66**, o1863. 10.1107/S1600536810024347 PMC3007065 21588060

[bb15] Zukerman-Schpector, J., Olivato, P. R., Cerqueira Jr, C. R., Santos, J. M. M., Ng, S. W. & Tiekink, E. R. T. (2011). *Acta Cryst.* E**67**, o2759. 10.1107/S1600536811037111 PMC3201309 22065241

